# The burden of premature mortality in Spain using standard expected years of life lost: a population-based study

**DOI:** 10.1186/1471-2458-11-787

**Published:** 2011-10-11

**Authors:** Ricard Gènova-Maleras, Ferrán Catalá-López, Nerea Fernández de Larrea-Baz, Elena Álvarez-Martín, Consuelo Morant-Ginestar

**Affiliations:** 1 Primary Care General Directorate, Regional Health Council, Madrid, Spain; 2 Centro Superior de Investigación en Salud Pública (CSISP), Valencia, Spain; 3 Fundación Instituto de Investigación en Servicios de Salud, Valencia, Spain; 4 Health Technology Assessment Unit, Laín Entralgo Agency, Regional Health Council, Madrid, Spain; 5 Department of Preventive Medicine and Public Health, Rey Juan Carlos University, Madrid, Spain; 6 Spanish National Drugs Strategy, Spanish Ministry of Health, Social Policy and Equality, Madrid, Spain; 7 Department of Health Information Systems, Regional Health Council, Madrid, Spain

## Abstract

**Background:**

Measures of premature mortality have been used to guide debates on future health priorities and to monitor the population health status. Standard expected years of life lost (SEYLL) is one of the methods used to assess the time lost due to premature death. This article affords an overview of premature mortality in Spain for the year 2008.

**Methods:**

A population-based study was conducted estimating SEYLL by sex and age groups. SEYLL, a key component of the disability-adjusted life years measure of disease burden, was calculated using Princeton West standard life tables with life expectancy at birth fixed at 80 years for males and 82.5 years for females. Population data and specific death records were obtained from the official registers of the National Institute of Statistics. All data were analysed and prepared in GesMor and Epidat software packages.

**Results:**

The burden of premature mortality was estimated at 2.1 million SEYLL when age at death is taken into account. Males lost 60.9% and females lost 39.1% of total SEYLL. Malignant tumors (34.5%) and cardiovascular diseases (24.0%) were the leading categories in terms of SEYLL. Ischaemic heart disease (8.5%) and lung cancers (8.0%) were the most common specific causes of SEYLL followed by cerebrovascular diseases (5.9%), colorectal cancer (4.1%), road traffic accidents (3.5%), Alzheimer and other dementias (2.9%), chronic obstructive pulmonary disease (2.8%), breast cancer (2.8%) and suicides (2.6%).

**Conclusions:**

In Spain, premature mortality was essentially due to chronic non-communicable diseases. Data provided in this study are relevant for a more balanced health agenda aimed at reducing the burden of premature mortality. This study also represents a first step in estimating the overall burden of disease in terms of premature death and disability.

## Background

Historically, mortality rates have been used to compare health status across populations. These measures do not fully account for the burden of premature mortality, an important indicator of a population health status. In fact, since most deaths occur among persons in older age groups, mortality rates are dominated by the underlying disease processes of the elderly [1]. Premature mortality entails estimating the average time a person would have lived had he or she not died prematurely. This estimation inherently incorporates age at death, rather than merely the occurrence of death itself [2].

In recent years, considerable efforts have been put into the development of summary measures of population health that combine information on mortality and non-fatal health outcomes. In the *Global Burden of Disease *study [3,4], a standardized form of years of life lost for measuring the burden of disease due to premature mortality was promoted. Particularly, the standard expected years of life lost (SEYLL) method was adopted to take into account the age at which deaths occur by giving greater weight to deaths occurring at younger ages and lower weight to deaths occurring at older ages. For several reasons, SEYLL has advantages when used as a summary measure of the burden of premature mortality, including: deaths at all ages contribute to the calculation of the burden of disease irrespective of the population in which they occur (in contrast with alternative approaches that use a fixed cut-off age e.g., 0-65, 0-75, or 1-70 years), as wells as deaths at the same age contribute equally to the burden of disease [5].

Health policy-makers face the challenge of responding to current disease prevention and control priorities, while being responsible for predicting future priorities. Ideally, such decisions should be based on summary measures of population health [6,7], including premature mortality. Summary measures are intended to guide debates on future health priorities, and they provide a way of monitoring and evaluating changes in the population health and the potential benefits of healthcare services [7].

In this context, the present article affords a detailed overview of the burden of premature mortality in Spain for the year 2008 by using SEYLL.

## Methods

The methods used are directly derived from those developed by Murray and Lopez in the *Global Burden of Disease *study [3]. We followed this methodology to quantify the magnitude and distribution of premature mortality in the Spanish population, by estimating SEYLL, one of the two components that summarize disability-adjusted life years (DALY), by cause, sex and age group for the year 2008. Following the disease burden classification system, diseases and injuries were presented into 3 broad groups: communicable diseases and maternal, perinatal and nutritional disorders (group I); chronic non-communicable diseases (group II); and all injuries (group III). These groups are divided into 21 major categories which can be disaggregated into approximately 100 subcategories.

SEYLL measure correspond to the number of unlived years in a population as a consequence of premature mortality and are calculated from the number of deaths multiplied by a standard life expectancy at the age at which death occurs. The formula for calculating SEYLL for a given cause is: SEYLL = KCe^ra^/(r+β)^2^[e^- (r + β)(L+a) ^[-(r+β)(L+a)-1] -e^-(r + β)a^[-(r+β)a-1]]+(1-K)/r(1-e^-rL^), where: r: discount rate (for health gains in the future); K: standard age-weighting modulation factor, a parameter that allows nonuniform (K = 1) age weighting to be used; C = constant = 0.1658; β = parameter from the age weighting function = 0.04; L = years of life left at age a; a = onset of disease year; e = constant = 2.71.

Data, both on the number of deaths by sex, age and specific causes and on the population for 2008 were obtained from the official registers of the National Institute of Statistics (*Instituto Nacional de Estadística*, INE) [8,9]. Individuals residing and dying of Spain in 2008 were considered in our analyses, with a mid-year population of 45,593,385 (22,512,354 males and 23,081,031 females). Anonymised death records are based on death certificates completed shortly after the time of death and are coded according to the International Classification of Diseases, 10^th ^Revision (ICD-10 codes). We applied a mortality standard norm based on a model life table (namely, Princeton West level 26 modified) which has a life expectancy at birth of 80 years for males and 82.5 years for females [9,10]. We used this standard life expectancy because is the same for deaths in all regions of the world and is the same as that used for the calculation of DALY measures. Additionally, social values were incorporated to the calculation of SEYLL, including 3% time discounting and non-uniform age weights that give less weight to years lived at early and older ages were used. We also followed the general approach proposed by Murray and Lopez to reclassify diagnoses for the ill-defined causes of death when needed.

All data were analysed and prepared in GesMor (Instituto de Salud Carlos III, Madrid, Spain) [11] and Epidat 4.0 (Consellería de Sanidade, Xunta de Galicia, Spain and Pan American Health Organization, Washington D.C., USA) [12] software packages.

## Results

In Spain, the burden of premature mortality was estimated as a total of 2,099,291 SEYLL (39.1 per cent of them in females) in 2008, with SEYLL rates per 1,000 Spanish people of 46.0 (56.8 for males and 35.6 for females). Overall, chronic non-communicable diseases (group II) accounted for 83.4 per cent of the total number of SEYLL (87.7 per cent in females), accidents and injuries (group III) for 10.1 per cent (2.1 per cent in females), and communicable, maternal, perinatal, and nutritional conditions (group I) accounted the remaining 6.5 per cent (Table 1).

**Table 1:** Standard expected life years lost (SEYLL) by sex and broad cause group. Spain, 2008.

	Males			Females			Both sexes		
Cause group	Number	%	Rate per 1,000	Number	%	Rate per 1,000	Number	%	Rate per 1,000
**Group I**	82,398	6.4	3.7	53,588	6.5	2.3	135,986	6.5	3.0
**Group II**	1,030,563	80.6	45.8	720,160	87.7	31.2	1,750,723	83.4	38.4
**Group III**	164,918	12.9	7.3	47,663	5.8	2.1	212,582	10.1	4.7

**Overall**	**1,277,879**	**100**	**56.8**	**821,412**	**100**	**35.6**	**2,099,291**	**100**	**46.0**

Group I: Communicable, maternal, perinatal, and nutritional conditions; Group II: Noncommunicable diseases; Group III: Accidents and injuries.

Malignant tumors, which account for 34.5 per cent of the total SEYLL, are the major cause of premature mortality in Spain for 2008 (Table 2), followed by cardiovascular diseases (24.0 per cent) and unintentional injuries (7.1 per cent). These three disease categories account for 65.6 per cent of the total SEYLL lost in the Spanish population. Respiratory diseases (6.2 per cent), digestive diseases (5.6 per cent) and mental and neurological disorders (5.6 per cent) rank fourth, fifth and sixth, respectively.

**Table 2:** Standard expected life years lost (SEYLL) by sex and cause categories. Spain, 2008.

	Males				Females				Both sexes			
Cause categories	Number	%	Rank	Rate per 1,000	Number	%	Rank	Rate per 1,000	Number	%	Rank	Rate per 1,000
**Malignant tumors**	445,321	34.8	1	19.8	278,056	33.9	1	12.0	723,377	34.5	1	15.9
**Cardiovascular diseases**	289,506	22.7	2	12.9	214,585	26.1	2	9.3	504,091	24.0	2	11.1
**Unintentional injuries**	115,913	9.1	3	5.1	33,018	4.0	6	1.4	148,931	7.1	3	3.3
**Respiratory diseases**	85,763	6.7	4	3.8	44,307	5.4	4	1.9	130,071	6.2	4	2.9
**Digestive diseases**	76,276	6.0	5	3.4	41,339	5.0	5	1.8	117,615	5.6	5	2.6
**Mental and neurological conditions**	55,029	4.3	6	2.4	62,261	7.6	3	2.7	117,290	5.6	6	2.6
**Infectious and parasitic diseases**	41,303	3.2	8	1.8	22,552	2.7	7	1.0	63,855	3.0	7	1.4
**Intentional injuries**	49,005	3.8	7	2.2	14,645	1.8	11	0.6	63,651	3.0	8	1.4
**Diabetes mellitus**	19,466	1.5	10	0.9	20,146	2.5	8	0.9	39,612	1.9	9	0.9
**Respiratory infections**	21,257	1.7	9	0.9	15,050	1.8	10	0.7	36,307	1.7	10	0.8
**Genitourinary diseases**	18,497	1.4	12	0.8	17,001	2.1	9	0.7	35,498	1.7	11	0.8
**Perinatal conditions**	19,295	1.5	11	0.9	14,575	1.8	12	0.6	33,870	1.6	12	0.7
**Congenital anomalies**	13,473	1.1	13	0.6	12,131	1.5	13	0.5	25,604	1.2	13	0.6
**Endocrine and blood disorders**	10,892	0.9	14	0.5	11,009	1.3	14	0.5	21,901	1.0	14	0.5
**Other (benign) tumors**	10,681	0.8	15	0.5	9,446	1.1	15	0.4	20,127	1.0	15	0.4
**Musculoskeletal diseases**	4,260	0.3	16	0.2	7,457	0.9	16	0.3	11,717	0.6	16	0.3
**Skin diseases**	1,376	0.1	17	0.1	2,387	0.3	17	0.1	3,763	0.2	17	0.1
**Nutritional deficiencies**	542	0.0	18	0.0	722	0.1	18	0.0	1,264	0.1	18	0.0
**Maternal conditions**	0	0.0	-	0.0	689	0.1	19	0.0	689	0.0	19	0.0
**Oral conditions**	19	0.0	19	0.0	32	0.0	20	0.0	51	0.0	20	0.0
**Sense organ diseases**	4	0.0	20	0.0	3	0.0	21	0.0	7	0.0	21	0.0

**Overall**	**1,277,879**	**100**		**56.8**	**821,412**	**100**		**35.6**	**2,099,291**	**100**		**46.0**

**Figure 1:**
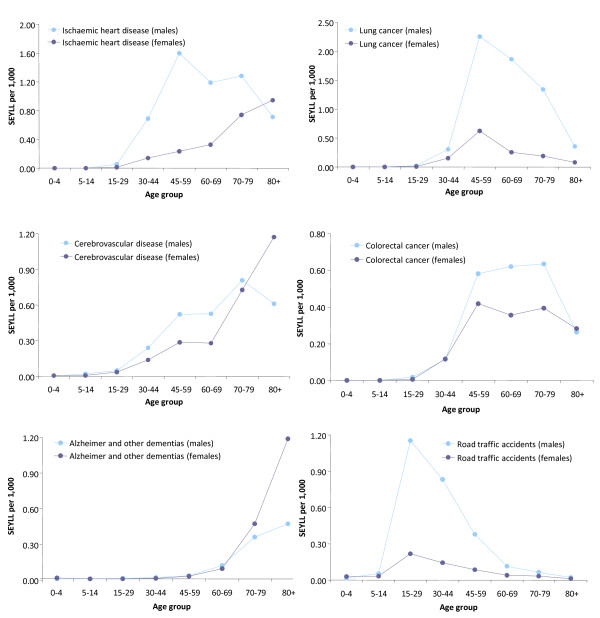
**Distribution of standard expected life years lost (SEYLL) rates by sex and age group among the leading specific disease conditions**.

Table 3 depicts the leading specific subcategories that caused most SEYLL. Ischaemic heart disease (8.5 per cent) and lung cancer (8.0 per cent) ranked first and second respectively, followed by cerebrovascular disease (5.9 per cent), colorectal cancer (4.1 per cent), road traffic accidents (3.5 per cent), Alzheimer and other dementias (2.9 per cent), chronic obstructive pulmonary disease (2.8 per cent), breast cancer (2.8 per cent) and suicides (2.6 per cent). Calculating SEYLLs by sex revealed males and females variations in the burden of premature mortality. The ranking of the leading disease categories and specific causes in terms of premature deaths was different for both sexes. Lung cancer (10.8 per cent) ranked first in males, followed by ischaemic heart disease (9.7 per cent), cerebrovascular disease (4.9 per cent) and road traffic accidents (4.6 per cent). In females, cerebrovascular disease (7.5 per cent) and breast cancer (7.1 per cent) were the first and second leading causes of SEYLL respectively, followed by ischaemic heart disease (6.7 per cent) and Alzheimer and other dementias (4.7 per cent). Figure 1 shows the distribution of SEYLL rates (per 1,000 people) attributable to each of the leading disease conditions by sex and age group.

**Table 3:** Standard expected life years lost (SEYLL) by sex and leading specific subcategories. Spain, 2008.

	Males				Females				Both sexes			
Specific subcategories	Number	%	Rank	Rate per 1,000	Number	%	Rank	Rate per 1,000	Number	%	Rank	Rate per 1,000
**Ischaemic heart disease**	124,257	9.7	2	5.5	55,176	6.7	3	2.4	179,433	8.5	1	3.9
**Lung cancer**	138,406	10.8	1	6.1	30,283	3.7	6	1.3	168,690	8.0	2	3.7
**Cerebrovascular disease**	62,601	4.9	3	2.8	61,215	7.5	1	2.7	123,816	5.9	3	2.7
**Colorectal cancer**	50,190	3.9	5	2.2	36,261	4.4	5	1.6	86,451	4.1	4	1.9
**Road traffic accidents**	59,333	4.6	4	2.6	13,708	1.7	12	0.6	73,041	3.5	5	1.6
**Alzheimer and other dementias**	20,958	1.6	14	0.9	39,001	4.7	4	1.7	59,959	2.9	6	1.3
**Chronic obstructive pulmonary disease (COPD)**	46,284	3.6	6	2.1	12,765	1.6	14	0.6	59,049	2.8	7	1.3
**Breast cancer**	498	0.0	-	0.0	58,413	7.1	2	2.5	58,911	2.8	8	1.3
**Suicides**	43,391	3.4	7	1.9	12,191	1.5	15	0.5	55,581	2.6	9	1.2
**Cirrhosis of the liver**	36,062	2.8	8	1.6	11,742	1.4	17	0.5	47,804	2.3	10	1.0
**Stomach cancer**	25,233	2.0	9	1.1	14,470	1.8	10	0.6	39,703	1.9	11	0.9
**Pancreas cancer**	22,133	1.7	11	1.0	16,379	2.0	8	0.7	38,512	1.8	12	0.8
**Lower respiratory infections**	21,093	1.7	13	0.9	14,837	1.8	9	0.6	35,930	1.7	13	0.8
**Liver cancer**	23,918	1.9	10	1.1	9,000	1.1	22	0.4	32,917	1.6	14	0.7
**Lymphomas and multiple myeloma**	18,518	1.4	18	0.8	14,344	1.7	11	0.6	32,861	1.6	15	0.7
**Inflammatory heart diseases**	19,570	1.5	16	0.9	12,132	1.5	16	0.5	31,702	1.5	16	0.7
**Brain cancer**	16,898	1.3	20	0.8	11,440	1.4	18	0.5	28,338	1.3	17	0.6
**Leukaemias**	14,516	1.1	22	0.6	10,593	1.3	20	0.5	25,110	1.2	18	0.6
**Human immunodeficiency virus/acquired immunodeficiency syndrome (HIV/AIDS)**	19,085	1.5	17	0.8	5,861	0.7	24	0.3	24,946	1.2	19	0.5
**Bladder cancer**	20,789	1.6	15	0.9	4,132	0.5	29	0.2	24,922	1.2	20	0.5

**Overall**	**1,277,879**	**100**		**56.8**	**821,412**	**100**		**35.6**	**2,099,291**	**100**		**46.0**

Note: In males, prostate cancer ranks 12^th ^(21,633 SEYLL), mouth cancer ranks 19^th ^(17,386 SEYLL) and poisonings ranks 21^st ^(16,883 SEYLL). In females, ovarian cancer ranks 7^th ^(17,026 SEYLL), hypertensive heart disease ranks 13^th ^(13,082 SEYLL), and nephritis/nephrosis ranks 19^th ^(10,968 SEYLL).

**Figure 2:**
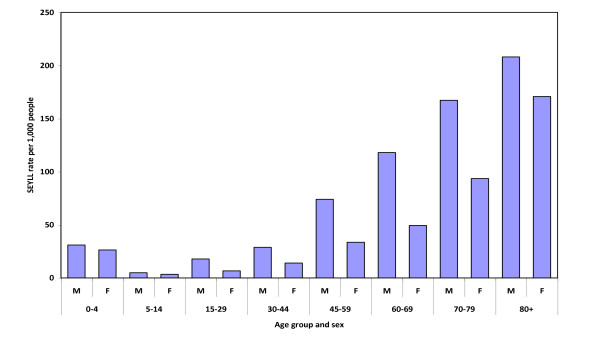
**Standard expected life years lost (SEYLL) rates by sex and age group. Spain, 2008**.

In terms of age, 39.8 per cent of the burden of premature mortality was concentrated in subjects aged over 70 years old, with the general trend being for SEYLL to rise with age (Table 4). Figure 2 shows the SEYLL rates (per 1,000 people) by sex and age group. A breakdown by subcategory revealed perinatal conditions (48.5 per cent) and congenital anomalies (25.3 per cent) as being important in the 0-4 years age group, and unintentional injuries (23.8 per cent), malignant tumors (22.2 per cent) and neurological and mental disorders (11.4 per cent) in the 5-14 years age group. Between the ages of 15 and 29 years, unintentional injuries continued as the leading cause of SEYLL (41.8 per cent) followed by malignant tumors (14.7 per cent), and there was a sharp rise in intentional injuries (14.0 per cent). In the 30-44 years age group, the premature deaths attributed to malignant tumors (27.5 per cent), unintentional injuries (18.7 per cent) and cardiovascular diseases (15.0 per cent) are worth noting. From 45 to 69 years, malignant tumors (49.5 per cent) assumed extremely important, particularly in the case of lung cancer (13.8 per cent), and cardiovascular diseases (21.0 per cent) such as ischaemic heart disease began to be relevant (9.2 per cent). Finally, among subjects aged over 70 years, cardiovascular diseases (33.9 per cent) and malignant tumors (27.4 per cent) were the first and second leading causes of SEYLL, respectively.

**Table 4:** Standard expected life years lost (SEYLL) by sex and age group. Spain, 2008.

	Males			Females			Both sexes		
Age group	Number	%	Rate per 1,000	Number	%	Rate per 1,000	Number	%	Rate per 1,000
0-4	38,158	3.0	31.0	30,540	3.7	26.3	68,698	3.3	28.7
5-14	10,768	0.8	4.9	7,073	0.9	3.4	17,841	0.8	4.1
15-29	79,649	6.2	17.9	27,982	3.4	6.7	107,631	5.1	12.4
30-44	171,202	13.4	28.8	79,022	9.6	14.0	250,224	11.9	21.6
45-59	320,571	25.1	74.0	148,153	18.0	33.6	468,724	22.3	53.6
60-69	240,286	18.8	118.0	110,362	13.4	49.4	350,648	16.7	82.1
70-79	259,726	20.3	167.3	185,257	22.6	93.5	444,983	21.2	125.9
80+	157,518	12.3	208.0	233,024	28.4	170.8	390,542	18.6	184.1

**Overall**	**1,277,879**	**100**	**56.8**	**821,412**	**100**	**35.6**	**2,099,291**	**100**	**46.0**

Detailed information on SEYLL by sex and age group for all disease and injury categories is given in additional file 1 ("Detailed information of SEYLL. Spain, 2008").

## Discussion

This study presents an estimation of the burden of premature mortality for diseases and injuries in Spain, largely on the basis of the SEYLL measure as developed in the *Global Burden of Disease *study. Our results clearly indicate the public health relevance of specific disease conditions and injuries in terms of premature and avoidable deaths. We estimated the burden of premature mortality in Spain at 2.1 million SEYLL in 2008. This figure represents 46.0 years of early deaths were lost among each 1,000 people. The Spanish male rate of 56.8 years, and female rate of 35.6 years per 1,000 population in 2008 compare well with other developed countries [4]. In the country, the leading causes of death when age at death is taken into account through SEYLL were chronic non-communicable diseases. Similarly, the greatest number of SEYLL was attributed to malignant tumors and cardiovascular diseases. In terms of specific causes, the burden of premature mortality in Spain was highest for ischaemic heart disease, followed by lung cancer and cerebrovascular disease, like in other developed and developing countries [4,13-15] and the corresponding SEYLL rates were lower even when compared with EURO-A subregion (e.g., 7.3, 3.9 and 3.7 per 1,000 people, respectively) [4]. For the unintentional injuries subcategory, however, other developed countries performed better than Spain for both males and females [4]. This subgroup of injuries include such causes of death as road traffic accidents, poisonings and accidental falls, among others.

A coordinated effort by health policy-makers is warranted against premature and preventable death from chronic non-communicable diseases by supporting prevention, control and rigorous evidence-based medical technologies and health policy or social programmes in particular areas. There are many important ways of achieving a more effective disease management: applying clinical guidance and consensus, reducing social inequalities by focusing further on the most disadvantaged groups (including those with a lower educational level), reinforcing action on the improvement of lifestyle factors (e.g., weight loss, alcohol consumption, cigarette smoking, and dietary salt intake), and intensifying the treatment (not only by drug therapies) in patients with uncontrolled risk factors such as obesity, smoking or arterial hypertension, among others [16]. In Spain, for example, the National Quality Plan for the National Health System [17] has developed health strategies to ensure highest quality in healthcare services elaborating national disease control strategies focusing on the most burdensome conditions in order to guarantee excellence in healthcare. Such approaches are advocating integrated focus combining disease prevention and treatment with the corresponding measures for cancer, ischaemic heart disease or cerebrovascular disease [18-24]. It is noteworthy to mention the fact that even though unintentional injuries (including road traffic accidents) are among the leading causes of SEYLL in Spain, most of the national research resources allocated have gone to heart and cancer control programmes in the past [25]. On this regard, there has been much less attention paid to the reduction of premature deaths in the injury, with no formal national disease prevention strategy in place.

The analysis presented in this article provide a framework for more detailed analysis of the national burden of disease, for a baseline against which the impact of the health status of population in Spain can be evaluated in a more complete way by means of generalised cost-effectiveness analyses of alternative disease control strategies. In the same way, previous SEYLL estimates have been helpful when identifying priority diseases for health research funding [25,26].

Calculating SEYLL need to have reliable sources of mortality data. Death records were based on nationwide registers from the National Institute of Statistics to measure the number and causes of deaths in the country. The quality and validity of mortality data depends mostly of the quality and accuracy of death certification. On this regard, we used classical algorithms developed in the *Global Burden of Disease *study [3,4] for correcting misclassification of deaths due to ill-defined causes when needed. Although local studies of the quality of the causes of death showed that mortality statistics in Spain appear to be reasonably reliable for being used for research and administrative purposes [27,28], recent interesting proposals for enhancing the algorithms of national causes of death data could have been included in our corrections [29,30]. Another of the limitations are the controversial social values incorporated into the SEYLL. With regard to the standard life expectancy, we decided to use a model life table [3,4] because it assigns life expectancy at all ages and gives consideration to high life expectancies. As the standard used in most burden of disease studies providing SEYLL, it also allows for comparisons to be made. One can argue for the use of country-specific life expectancies as an age limit adjusts the premature mortality calculation to the population profile of the country or area. However, the problem with this approach is that the premature mortality will not be comparable with that of other populations with different life expectancies.

Another limitation of this study is that the SEYLL measure we applied is strongly influenced by death rates and do not provide a full picture of how different disease conditions affect population health [6]. Classical methods of years of life lost take into account only death at younger ages and decrease the burden of premature death more if mortality is lower and occurs at older ages. Given that a life table standard with a very low mortality (as applied in the SEYLL method proposed in the *Global Burden of Disease *study) increases relatively the burden of mortality at older ages [31], other summary measures of population health status such as DALYs and quality-adjusted life years (QALY) are therefore receiving increasing prominence. These measures combine information not only on the duration of life, but also on non-fatal disabling outcomes, and may encompass morbidity, disability and mortality outcomes in a single metric. Along these lines, SEYLL reported here represents a first step in estimating the overall burden of disease in terms of premature death and disability. Our estimates could be improved in the future, for example, by collecting incidence and disease duration data at a national population-level and calculating the DALY metric.

## Conclusions

Calculating SEYLL specifically for Spain provides a systematic analysis of premature deaths at a population level and is an important component for identifying disease areas where greater attention by health policy-makers is required because they represent preventable loss of life. Burden of premature mortality in Spain was fundamentally attributable to chronic non-communicable diseases, specifically malignant tumors, cardiovascular diseases, unintentional injuries, respiratory diseases, digestive diseases and mental and neurological conditions. We hope the results of this study will lead to a more balanced health agenda in Spain, with adequate investment in health research, training activities, and intervention programs aimed at reducing the burden of premature mortality.

## Competing interests

The authors declare that they have no competing interests.

## Authors' contributions

RGM conceived the study aims and design, and developed the study in discussions with FCL, EAM, CMG and NFL. All authors contributed to interpretation of results, revised and commented on the manuscript for important intellectual content, and approved the final version.

## Pre-publication history

The pre-publication history for this paper can be accessed here:

http://www.biomedcentral.com/1471-2458/11/787/prepub

## Supplementary Material

Additional file 1 **Detailed information of SEYLL. Spain, 2008**. We provided detailed tabulations of SEYLL for the disease categories and specific causes included in Spanish National Burden of Disease study (mortality premature component), classified by disease groups, age, and sex. Click here for file
